# Fungal Virus, FgHV1-Encoded p20 Suppresses RNA Silencing through Single-Strand Small RNA Binding

**DOI:** 10.3390/jof8111171

**Published:** 2022-11-07

**Authors:** Shuangchao Wang, Jingze Zhang, Clement Nzabanita, Mingming Zhang, Jianhua Nie, Lihua Guo

**Affiliations:** 1 State Key Laboratory of Plant Diseases and Insect Pests, Institute of Plant Protection, Chinese Academy of Agricultural Sciences, Beijing 100193, China; 2 Functional and Evolutionary Entomology, Gembloux Agro-Bio Tech, University of Liège, 5030 Gembloux, Belgium

**Keywords:** fungal virus, RNA silencing, FgHV1, p20, RNA silencing suppressor, single-strand small RNA binding

## Abstract

Fungal viruses are widespread in fungi infecting plants, insects and animals. High-throughput sequencing has rapidly led to the discovery of fungal viruses. However, the interactive exploration between fungi and viruses is relatively limited. RNA silencing is the fundamental antivirus pathway in fungi. *Fusarium graminearum* small RNA (sRNA) pattern was regulated by Fusarium graminearum hypovirus 1 (FgHV1) infection, indicating the activation of RNA silencing in virus defense. In this study, we focused on the function of an uncharacterized protein sized at 20 kD (p20) encoded by FgHV1. In the agro-infiltration assay, p20 was identified as a novel fungal RNA silencing suppressor. p20 can block systemic RNA silencing signals besides local RNA silencing suppression. We further elucidated the RNA silencing suppression mechanism of p20. The single-strand sRNA, instead of double-strand sRNA, can be incorporated by p20 in electrophoretic mobility shift assay. p20 binds sRNA originating from virus and non-virus sources in a non-sequence-specific manner. In addition, The *F. graminearum* 22 and 23-nt sRNA abundance and pathways related to RNA processing and redox regulation were regulated by p20. Our study revealed the first fungal virus-encoded RNA silencing suppressor with sRNA binding capability.

## 1. Introduction

A fungal virus (mycovirus) is a kind of virus that replicates inside fungi [1]. The genome of fungal viruses exists in the form of single, double-strand RNA, and single-strand DNA [2]. Based on the latest report of the International Committee on Taxonomy of Viruses, fungal viruses are classified into 23 families (https://ictv.global/, accessed on 14 October 2022). Among them, sense single strand RNA fungal viruses are the largest group. Most fungal viruses exist as latent or asymptomatic infections. With the development of high-throughput sequencing technology, fungal viruses have been excavated and characterized more rapidly. Currently, some fungal viruses have the ability to attenuate the pathogenicity of host fungi, such as *Cryphonectria parasitica*, *Sclerotinia sclerotiorum* and *Fusarium graminearum* [3,4,5,6]. It is worth noting that Sclerotinia sclerotiorum hypovirulence-associated DNA virus 1 can convert a pathogenic host to endophytic fungi, which can promote crop production and induce disease resistance [7].

*Fusarium* spp. Causing Fusarium head blight threatens wheat, barley and small grain cereals worldwide [8]. More than 20 viruses have been sequenced from *Fusarium* spp [9]. Among them, Fusarium graminearum hypovirus 1 (FgHV1), sized at 13,023 nt, is the first hypovirus discovered from *F. graminearum*. FgHV1 is closely related to Cryphonectria hypovirus 1 (CHV1) [10]. However, their virulence on host pathogenicity is quite different. CHV1 caused serious effects on *S. sclerotiorum* morphology, pathogenicity, pigmentation and sporulation, while FgHV1 had minor effects on fungal morphology, growth and spore production [3,10]. Additionally, 378 genes are differentially regulated upon FgHV1 infection in *F. graminearum*. These genes are mainly related to transcription factors, cellular redox regulation and ubiquitination system. Moreover, p20 encoded by FgHV1 can induce hyper antisense response and H_2_O_2_ accumulation in *Nicotiana benthamiana* leaves [11].

RNA silencing is the fundamental pathway for gene expression regulation and virus defense [12]. RNA silencing can be divided into transcriptional gene silencing (TGS) and post-transcriptional gene silencing (PTGS). In fungi, plants and animals, the critical components and sRNA processing flow are relatively conserved for PTGS. Briefly, innate or virus-originated dsRNA was cleaved by Dicer (Dcl) into 20–30 nt small RNA (sRNA). Then sRNA was loaded into Argonaute (Ago), forming an RNA-induced silencing complex (RISC), and directing target gene cleavage. The RNA silencing signal can be amplified by RNA-dependent RNA polymerase (RdRp) [13]. Small interfering RNA (siRNA) is the core molecular during the RNA silencing process, and its 3′ end has two hanging bases. According to the different sources of siRNA, it can be divided into endogenous siRNA and exogenous siRNA. Endogenous siRNA is the RNA transcribed from DNA in an organism, which is processed by Dcl digestion. Exogenous siRNA is the siRNA from the virus after the virus infects an organism. At present, phase siRNA (phasiRNA) is the most widely studied siRNA [14]. phasiRNA is a major subclass of plant secondary siRNA, which is mediated by microRNA (miRNA) and has the characteristics of regular spacing. We characterized the small RNA (sRNA) in FgHV1 infected *F. graminearum*, and it was revealed that there were 1,831,081 (88,911 unique) sRNA reads mapped to FgHV1 genome, accounting for 16.51% of *F. graminearum* total sRNA [15]. In addition, the sRNA length distribution and host miRNA were also influenced. These results indicate that RNA silencing was activated and occupied as an important antivirus defense mechanism upon FgHV1 infection.

To counter-dense host RNA silencing, viruses encode various RNA silencing suppressors (RSS) [16]. Until now, four RSS have been characterized by fungal viruses. CHV1 encoded p29 can suppress the expression of RNA silencing critical genes, *CpDCL2* and *CpAGO2* [17,18,19]. Similarly, *CpDCL2* was also suppressed by p24 from CHV4 [20]. By binding to promoter regions of *FgDICER2* and *FgAGO1*, the ORF2 protein of Fusarium graminearum virus 1 protect the virus from host RNA silencing elimination [21]. For the fourth RSS, s10 of Rosellinia necatrix hypovirus 2, the RNA silencing mechanism is unknown [22]. To explore the function of FgHV1 encoded p20 during RNA silencing, we identified p20 as a novel fungal virus RSS and elucidated its RNA silencing mechanism in this paper.

## 2. Materials and Methods

### 2.1. Fungi and Plants Cultivation

The cDNA of p20 were reverse transcripted and amplified from RNA extracted from FgHV1 infected *F. graminearum* strain 4, which was collected and kept in our lab. After sequencing, the fragment was inserted into multiple clone sites (MCS) of PGTN vector for fungal transformation. In the agro-infiltration assay, p20 was inserted into pGD construct for transient expression. P19-pGD (protein p19 encoded by Carnation Italian Ring-spot Virus) and GFP-pGD construct were used and kept in our laboratory.

*F. graminearum* PH-1 strain, stored in our laboratory, was used for constructs transformation. All fungal strains were maintained at 4 °C and −80 °C in 25% glycerin. The growth condition of *F. graminearum* was at 25 °C in the dark on the PDA plate. The fresh mycelium used for DNA or RNA extraction were obtained from 3-day-old cultures grown on PDA overlaid with cellophane membrane (Promega, WI). *N. benthamiana* wild type and transgenic *N. benthamiana* (line 16 c) seeds were a gift of Dawei Li from China Agricultural University. Plants were grown in a greenhouse at 25 °C with 16/8 light and dark cycles. Four-week-old plants were used for agro-infiltration.

### 2.2. sRNA Sequence Used for sRNA Binding and Chemical Synthesis

The sequence of 21 nt eGFP-sense small single-strand RNA was 5′-GCUGACCCUGAAGUUCAUCUU-3′. 21 nt eGFP-antisense small single-strand RNA was 5′-GAUGAACUUCAGGGUCAGCUU-3′. 24 nt eGFP-sense small single-strand RNA was 5′-CGUACGCGGAAUACUUCGAAAGUU-3′. 24 nt eGFP-antisense small single-strand RNA was 5′-CUUUCGAAGUAUUCCGCGUACGUU-3′. The sense and antisense single-strand sRNA were designed as complementary with a 2 nt 3′ overhang for duplex formation. Double-strand small RNA was synthesized with the above sense and antisense single-strand small RNA. The concentration of single-strand small RNA was 100 μM, and the reaction mix together with 5× annealing buffer (Takala, Japan) was denaturated at 94 °C for 5 min and then incubated at 37 °C for 1 h. The FgHV1 derived sRNA for specificity binding test was siRNA-t1130926 (5′-CTCCAGTAGCATGTTCTTCGT-3′) targeting FGSG_09025 (Dicer 1), siRNA-t0999532 (5′-CTCCAGTAGCATGTTCTTCGTG-3′), targeting FGSG_09025 (Dicer 1) and siRNA-t0031901 (5′-TGGAAGAGAATGACGATATT3′), targeting FGSG_09076 (RDRP 5).

### 2.3. Agro-Infiltration-Mediated Technique for RNA Silencing Suppressor Identification

p20-PGD, p19-PGD, GFP-PGD and pGD were transformed into *Agrobacterium tumefaciens* (strain EHA105) with electroporation. An equal volume of p20-PGD, p19-PGD (positive control) and pGD (negative control) transformed *A. tumefaciens* were mixed with *A. tumefaciens* containing GFP-PGD. All *A. tumefaciens* strains were adjusted to OD600 = 1 before mixing. The mixtures were stood for at least 3 h before infiltration. The middle leaves of 4-week-old wild type or GFP transgenic *N. benthamiana* were injected with 1 ml of the mixture with sterile syringes. The injection point was in the middle of the leaf in half. The GFP fluorescence was checked by handheld UV light. Leaves and plants were photographed at 3 and 15 dpi. Each agro-infiltration experiment was conducted on at least 20 leaves of 10 plants and repeated three times.

### 2.4. p20 Expression and EMSA Assay

p20 gene was reverse transcribed and PCR amplified from FgHV1 genome RNA. The segment was purified and cloned into MCS sites of the protein expression vector, pET30 a in *Escherichia coli* BL21, tagged with histidine. The transformed BL21 strain was cultured in LB medium with 1 mM IPTG at 16 °C for 16 h. Then the bacteria cells were collected by centrifugation. The his-tagged protein was purified with affinity chromatography by ÄKTA. p20 purity was separated on 12% polyacrylamide gel electrophoresis and stained with coomassie blue dye. The final protein was adjusted to 1 mg/mL for electrophoretic mobility shift assay (EMSA), and the reaction concentration of chemically synthesized siRNA was 100 μM. Four EMSA reactions were conducted with 0, 1, 3, 9 μL of p20, respectively. The reaction mix was incubated at room temperature for 30 min and loaded in 8% native polyacrylamide gel for electrophoresis. The gel was run for 10 min at 30 mA, followed by 40 min at 10 mA. The gel was transferred for nucleic acid and protein staining, respectively, using an EMSA kit (Invitrogen, Leiden, The Netherlands).

### 2.5. Small RNA Sequencing and Analysis

Total RNA was extracted, and quality was determined by HPLC. Additionally, the qualified RNA was used for high-throughput sequencing using the DNBSEQ-T7 system (MGI Tech, China). The original data was obtained by sequencing (Raw Reads); before data analysis was carried out, low-quality data and adapters were removed. By mapping the sequencing data with the reference genome sequence, the contamination or confusion in the sample was judged. Samples with very high genome mapping rates were used for the following analysis. Three databases were used for non-coding RNA identification. miRBase V22 (http://www.mirbase.org/, accessed on 14 October 2022) database contain a large number of miRNA information and target gene of animals and plants. RNAcentral V16.0 (https://rnacentral.org/, accessed on 14 October 2022) is a non-coding RNA database, and it provides the most comprehensive and up-to-date non-coding RNA information on different species. The Rfam V13.0 database (https://rfam.xfam.org/, accessed on 14 October 2022) is a collection of RNA families, each represented by multiple sequence alignments, consensus secondary structures and covariance models. Based on the taxonomy ID of the species, we downloaded the non-coding RNA sequence of this species from miRBase and RNAcentral, and then map reads to these sequences to identify known non-coding RNAs. After these, the unannotated reads were mapped to Rfam. However, if there were no non-coding RNA in miRBase and RNAcentral, reads were mapped to Rfam directly. Based on the AASRA theory (http://biorxiv.org/content/early/2017/05/01/132928, accessed on 14 October 2022), we chose the best mapping of each read, and then stat the TPM (tags per million reads) expression of each non-coding RNA (more information of TPM, please see the help document of this report). After obtaining the expression of all non-coding RNAs, principal component analysis and correlation analysis were carried out. According to the results of correlation analysis and principal component analysis, the similarity between samples can be judged. The better the repeatability, the greater the correlation coefficient, and the closer the cluster in the principal component analysis diagram. The sRNA sequencing raw data was submitted to the national center for biotechnology information website (https://www.ncbi.nlm.nih.gov/, accessed on 14 October 2022) under project No. PRJNA879941.

### 2.6. Transcriptome Profiling

RNA for transcriptome sequencing was prepared as follows: mRNA was enriched by oligodT beads. rRNA was eliminated by RNase H digestion after hybridization with a DNA probe. The probe was digested with DNase. The RNA was cut into appropriate lengths and reverse transcripted with a random N6 primer. Then the second strand was synthesized. The products were phosphorylated and ligated with an adapter. After amplification, the heated single strand was cyclized and sequenced. The clean reads were mapped to *F. graminearum* PH-1 genome with Bowtie2. v1.2.8 (http://deweylab.biostat.wisc.edu/rsem/rsem-calculate-expression.html, accessed on 14 October 2022). DEseq2 was used for differentially expressed gene analysis with *p* ≤ 0.05. Based on GO and KEGG annotation, differentially expressed genes (DEGs) were enriched into different terms with pHYPER function in R statistical software. The RNA sequencing raw data was submitted to the national center for biotechnology information website (https://www.ncbi.nlm.nih.gov/, accessed on 14 October 2022) under project No. PRJNA879941.

## 3. Results

### 3.1. FgHV1 Encoded p20 Functions as a Fungal RNA Silencing Suppressor

**Figure 1:**
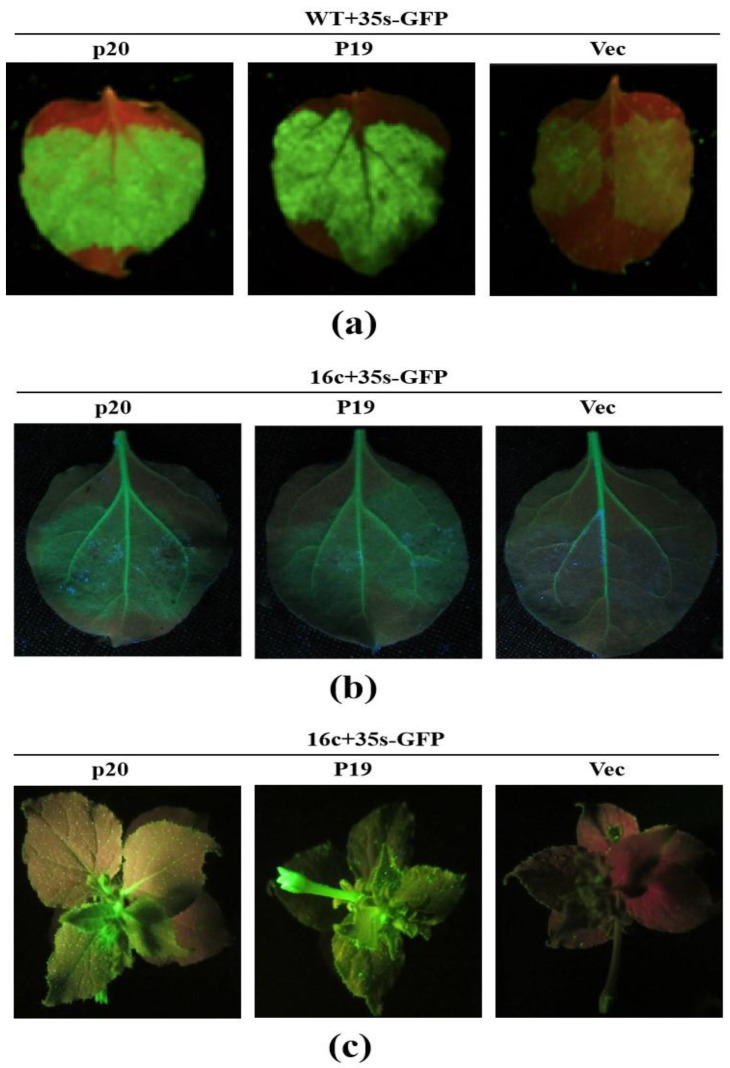
Local and systemic silencing suppression mediated by FgHV1-encoded p20. Leaves of *N. benthamiana* wild type (**a**) and GFP transgenic *N. benthamiana* 16 c line (**b**,**c**) co-infiltrated with *Agrobacterium tumefaciens* mixture containing pGD-GFP and RSS candidate, p19, p20 and Vec. In *N. benthamiana* wild type infiltration, green fluorescence was observed at 3 dpi on infiltrated leaves. In GFP transgenic *N. benthamiana* 16 c line, GFP signals were photographed under handheld UV light 3 dpi and 15 dpi on local infiltrated and systemic newly emerging leaves, respectively. WT (*N. benthamiana* wild type), 16 c (GFP transgenic *N. benthamiana* 16 c line), 35 S-GFP (*A. tumefaciens* containing pGD-GFP construct for GFP expression), p19 (*A. tumefaciens* containing pGD-p19, positive control), p20 (*A. tumefaciens* containing pGD-p20), Vec (*A. tumefaciens* containing pGD empty vector, negative control).

In an attempt to identify a potential RNA silencing suppressor encoded by fungal virus, FgHV1, the mature GFP silencing suppression system used for many plant and animal virus RSS identifications systems, was used [23]. Binary vectors pGD containing p19 of Carnation Italian Ring-spot Virus (CIRV) and p20 of FgHV1, respectively, were constructed in this study. p19 is a strong viral silencing suppressor. pGD-p19 construct was used as a sense control and pGD empty vector as a negative control. *Nicotiana benthamiana* wild type was co-infiltrated with *Agrobacterium* strain containing pGD-GFP construct under 35 s promoter for GFP expression (35 s-GFP). After mix-infiltration with pGD-p19, p20 and empty vector, green fluorescence was still stable and clear in p20 and p19 co-infiltrated leaves at 3 dpi, while the GFP signal was disappearing in the empty control (Figure 1a). This result indicates that p20 can protect GFP from degradation in local infiltrated leaves.

To further examine the local suppression of the RNA silencing capability of p20, RSS candidates together with a sense GFP transgene (35 s-GFP), RNA silencing inducer, were transiently co-expressed in GFP-transgenic *N. benthamiana* line 16 c (16 c) [24,25]. The sense GFP expression in 16 c induces RNA silencing of itself and the 16 c GFP transgene. p20, p19 and empty vector construct transformed agrobacteria were mixed with GFP expressing agrobacteria under a 3:1 ratio prior to agro-infiltration, respectively. One-month-old *N. benthamiana* line 16 c were agro-infiltrated with the three mixture groups, respectively. At 3 dpi, the agro-infiltrated leaves were observed for green fluorescence under a handheld UV trigger with an orange filter. As shown in Figure 1b, green fluorescence was not observed on leaves agro-infiltrated with a pGD empty vector together with 35 s-GFP expressing constructs, and a red patch developed in agro-infiltrated 16 c leaves, demonstrating that 35 s-GFP construct was not expressed efficiently in GFP-transgenic *N. benthamiana* plant as a result of RNA silencing stimulating. In contrast, the RNA silencing suppressor, p19 construct agro-infiltrated leaves exhibited obvious green fluorescence. The green fluorescence intensity was also obvious on p20 agro-infiltrated leaves, which indicates that p20 protect GFP from degradation by suppressing the host RNA silencing system. This phenomenon supported that p20 inhibited local RNA silencing on infiltrated leaf portions. 

RNA silencing signal can move systemically to other tissues in an organism [26]. Next, we examined whether p20 can inhibit systemic RNA silencing. GFP silencing signal was transmitted from 35 s-GFP construct agro-infiltrated leaf to the newly grown upper leaves in 16 c. At 15 dpi, 80% of the newly emerging leaves showed red veins developing in empty vector and 35 s-GFP constructs mixed agro-infiltrated 16 c plants, suggesting systemic RNA silencing. On the contrary, green fluorescence on newly emerging leaves was strong in p19 mix-infiltrated plants, indicating that p19 (positive control) inhibits the systemic RNA silencing signal transmission from lower co-infiltrated leaves to upper leaves. As to p20 co-infiltrated with 35 s-GFP on lower 16 c leaves, strong green fluorescence can still be observed in most newly emerging leaves (Figure 1c). Altogether, these results demonstrated that p20 interfered with the long-distance spread of RNA silencing signal. To our knowledge, p20 is the first report of a fungal virus-encoded RNA silencing suppressor inhibiting systemic RNA silencing.

### 3.2. p20 Incorporates Small Single-Strand RNA Instead of Double-Strand RNA

**Figure 2:**
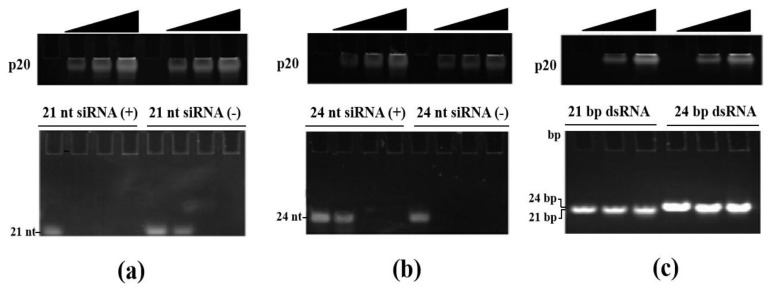
p20 incorporates single-strand small RNA instead of double-strand small RNA; 21 nt single-strand sense and antisense small RNA (**a**), 24 nt single-strand sense and antisense RNA (**b**), 21 bp and 24 bp double-strand RNA (**c**) were incubated with increasing amounts of p20. The upper gel showed protein visualization. The lower gel showed free small RNA staining in the same gel; 21 nt siRNA (+), (21 nt sense strand small interfering RNA); 21 nt siRNA (−), (21 nt antisense small interferingRNA); 24 nt siRNA (+), (24 nt sense strand small interferingRNA); 24 nt siRNA (−), (24 nt antisense small interferingRNA); 21 bp dsRNA, (21 bp double-strand RNA); 24 bp dsRNA, (24 bp double strand RNA).

sRNA plays a critical and central role in RNA silencing, which is produced by Dcl cleavage and then loaded into Ago, forming the RISC complex [27]. Virus infection triggers RNA silencing response and leads to sRNA accumulation. To counter-defense RNA silencing, some plant and animal virus RSS can suppress RNA silencing by sRNA incorporating. However, no fungal virus RSS have been reported with sRNA binding capability. To figure out the potential RNA silencing suppression mechanism of p20, we tested the p20 sRNA incorporating ability by EMSA. The EMSA is the most frequently used assay to detect RSS affinity for sRNA. Previously, we examined the sRNA profile of FgHV1 infected *F. graminearum* and found that 21 and 24-nt were the sRNA peaks and most regulated sRNA lengths. As it is demonstrated above, p20 suppresses GFP silencing in 16 c plants. Hence, 21 and 24-nt sense and antisense single-strand GFP RNA were chemically synthesized. In addition, 21 and 24 bp double-strand GFP RNA were acquired by single-strand RNA annealing from sense and antisense single-strand GFP RNA, respectively. In the binding reactions, 1 μL of sRNA (100 μM) was incubated with 0, 1, 3 and 9 μL of purified p20 (1 μg/μL). After incubation, p20 protein and sRNA were visualized on the same gel. If the sRNA were trapped in p20, the protein-sRNA complex moved much slower than free sRNA in gel. As for 21 and 24-nt sense and antisense single-strand sRNA binding, there were less free sRNA observed in gel with an increasing concentration of p20 (Figure 2a,b). All free single-strand sRNA bands disappeared when incubated with 3 μg of p20 (Figure 2a,b). We further investigated the sRNA binding capability of p20 with double-strand sRNA. In this experiment, dsRNA was also incubated with the same increasing concentration of p20 as with single-strand sRNA binding. When compared with the negative control, the free 21 and 24 bp double-strand showed nearly the same level after incubation with increasing concentration of p20, which indicated that p20 could not bind double-strand sRNA (Figure 2c). Collectively, these results suggested that p20 had the incorporating capability of single-strand sRNA instead of double-strand sRNA. This is the first report of fungal virus-encoded RSS with sRNA binding ability to suppress RNA silencing.

### 3.3. Influence of p20 on F. graminearum Small RNA Profiling 

**Figure 3:**
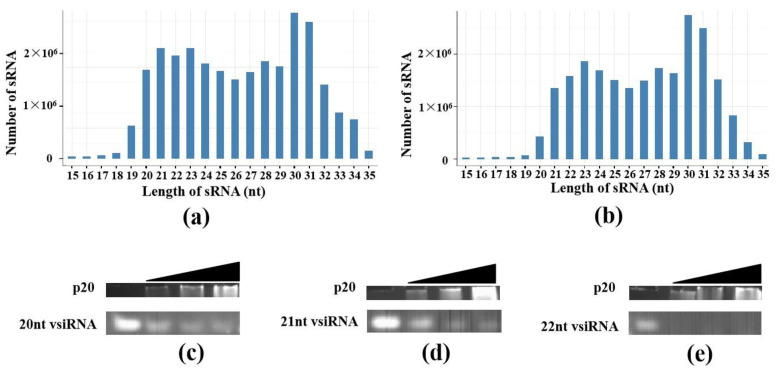
Influence of different sRNA length abundances by p20 in *F. graminearum* and non-sequence specific binding of p20. Length distribution and abundance of total sRNA in empty vector transformed *F. graminearum* (**a**) and p20 expressing vector transformed *F. graminearum* (**b**). sRNA were determined by high-throughput sRNA sequencing; 20, 21 and 22-nt single-strand virus-derived small RNA (vsiRNA) (**c**–**e**) was incubated with increasing amounts of p20, respectively. Protein concentration and free vsiRNA were visualized on the same gel with UV monitoring.

As demonstrated above, p20 had single-strand small RNA incorporating capability. Small RNA profiles of p20 transformed *F. graminearum* were examined via sRNA deep sequencing. In this paper, p20 and p20-r1 libraries were presented as two replicates of p20 transformed *F. graminearum* deep sequencing databases, while PGTN, PGTN-r1 libraries were two replicates of PGTN-empty vector transformed *F. graminearum* deep sequencing databases. After filtering low-quality data and adapters, the total sRNA clean reads number ranged between 22.84 to 24.61 M of each sample. In general, the length of micro-like RNA in p20 and the control group were found to be between 21–23 nt, piwi-interacting (piRNA) was found to be between 26–32 nt, small interfering RNA was found to be between 20–25 nt. The genome mapping rate ranged between 90.30 to 93.12%. The clean sRNA reads number for PGTN, PGTN-r1, p20 and p20-r1 was 23,772,921, 22,955,622, 22,844,246 and 24,607,579, respectively. The number of sRNA reads in the p20 group did not decline significantly. This indicated that p20 could not bind a large number of sRNA. Length distribution analysis allowed us to check the abundance of each sRNA length. Thus, we also analyzed the influence of p20 on the length distribution of sRNA in *F. graminearum*. As shown in Figure 3a,b, although the length distribution pattern of the two groups was similar, the abundance and relative proportion of 22 and 23-nt sRNA were different.

Further, we wondered if FgHV1-encoded p20 differs in its ability to bind exogenous and autologous sRNA (virus-originated and non-itself sRNA). Three virus-derived 20, 21 and 22-nt sRNA (vsiRNA) were synthesized and used for the protein-RNA binding capacity test. We also carried out EMSA to investigate whether they can be incorporated into p20. As shown in Figure 3c–e, as p20 concentration increased, 20, 21 and 22-nt virus-derived sRNA were all captured by p20. This result indicated that p20 bound sRNA in a non-sequence-specific manner without sRNA source selectivity.

### 3.4. F. graminearum Transcriptome Regulation by p20

**Figure 4:**
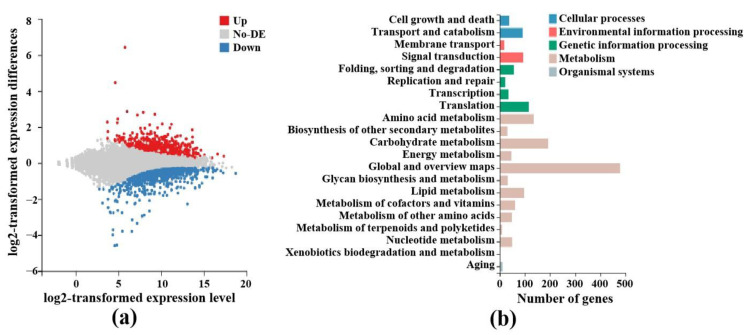
Differentially expressed genes and KEGG pathways regulated by p20 in *F. graminearum*. (**a**) p20 up- and down-regulated DEGs. The *x*-axis represented the A value (log2-transformed average expression level), and the *y*-axis represented the M value (log2-transformed multiples of difference). Red represents up-regulated DEGs, blue represents down-regulated DEGs, and gray represents non-DEGs. (**b**) p20 influenced enriched KEGG pathways. The *x*-axis was the number of genes annotated to a KEGG pathway category, and the *y*-axis was the KEGG pathway category.

To examine genes and pathways that were influenced by p20 in *F. graminearum* host, we examined the DEGs via RNA sequencing. Compared with the empty vector control, 1764 genes were differentially expressed upon p20 expression in *F. graminearum*, which accounted for about 10% of total host genes (Figure 4a). Among them, the highest expressed genes in p20 group were FGSG_04350 and FGSG_10996, whose functions were related to chitinase and ATP-dependent RNA helicase. It is known that FgHV1 also encoded an RNA helicase, which was important for RNA virus replication. The p20-induced host RNA helicase high expression may be employed by the virus for its replication. The largest down-expressed genes were FGSG_12523 and FGSG_06770. FGSG_12523 was involved in DNA repair, and FGSG_06770 was related to 5’-3’ exoribonuclease function. These two gene expressions suppressed by p20 may protect the FgHV1 genome from degradation and be beneficial for FgHV1 genome accumulation in the host. A KEGG pathway enrichment analysis of DEGs can help us to identify how genes interact and perform their roles in biological functions (Figure 4b). RNA processing pathways, including folding, sorting, degradation, replication, repair and nucleotide metabolism, were enriched. It is also worth noting that many genes, such as FGSG_09063 and FGSG_03749, were involved in redox regulation terms. This result suggests that p20 can not only bind sRNA but also regulate gene expression by other unexplored means.

## 4. Discussion

Fungal virus (mycovirus) exists in all kinds of fungi [28]. Mycovirus serves as a good tool for fundamental research of fungal biology and pathogenic fungi biocontrol application [29,30]. A total of 70–80% of plant diseases are caused by fungal infection [31]. The mycoviruses that are capable of attenuating the pathogenicity of host fungi attract more attention for their biological control potentiality. Many virulence-attenuating fungal viruses are classified into *Hypoviridae* family. FgHV1 is the first hypovirus, belonging to *Hypoviridae*, discovered in *F. graminearum* [10]. In the prior research, we studied the influence of FgHV1 on host RNA silencing and found that the sRNA length abundances were altered by FgHV1 infection [11,15]. Moreover, lots of FgHV1-derived sRNA were produced and distributed along the virus genome in a non-random pattern. To counter defense host RNA silencing, viruses also have different measurements to protect themselves from degradation. In this paper, we found that FgHV1-encoded p20 was an RNA silencing suppressor and elucidated its RNA silencing suppression mechanism. To the best of our knowledge, this is the first report of fungal virus-encoded protein with sRNA incorporating capability to suppress host RNA silencing.

Using the GFP-transgenic *N. benthamiana* line 16 c, our experiments demonstrated that the p20 expression construct could inhibit RNA silencing induced by GFP mRNA co-infiltration. In addition, we wondered whether p20 could suppress systemic RNA silencing signal transmission. It was quite exciting to find that the systemic RNA silencing signal was blocked by p20. In fungi, further experiments can be designed to confirm whether p20 can facilitate virus spread and benefit other viruses’ co-infection for its systemic RNA silencing suppression capability.

Further, we elucidated the RNA silencing suppression mechanism of p20. Based on protein structure, p20 might have sRNA-incorporating channels. EMSA indicated that single-strand sRNA could be loaded into p20, while double-strand sRNA cannot be incorporated inside. The mechanisms of RSS are also various in different viruses infecting plants and animals. Until now, there have been four fungal virus RSS, and three of them have been characterized by their suppression mechanism. p29 and p24 from CHV1 and CHV4 could interact with RNA silencing-related protein, thus suppressing the expression of RNA silencing critical genes, *FgDCL2* and *FgAGO2* [17,18,19,20]. In contrast, FgV1-encoded ORF2 had DNA binding capability [21]. The promoter region of *FgDCL2* and *FgAGO1* could be bounded by ORF2, which resulted in their expression suppression. Further experiments about other RSS mechanisms of p20 can be explored.

Because of the sRNA binding capacity of p20, it was reasonable to speculate that the total sRNA amount in the host would be reduced. Therefore, we sequenced the sRNA in p20-transformed *F. graminearum*. However, the total sRNA reads were nearly equal in p20 transformed and empty construct control groups. This result indicated that p20 could not bind a large number of sRNA in the host, even with sRNA binding ability. However, it was also possible that FgHV1 infection induces RNA silencing and sRNA production increase. Partial sRNAs were incorporated into p20, and then the accumulation of sRNA was returned to a normal level. We also analyzed the data in detail and found that 22 and 23-nt sRNA abundances were regulated. Then we were curious about the sequence selectivity of p20. It is well known that the targets of some virus-derived sRNA were host genes, which mediated host gene suppression. Two RNA silencing critical genes, *FgDicer1* (FGSG_09025) and *FgRdRp5* (FGSG_09076), have been predicted as targets of three FgHV1-derived sRNAs. The incorporation of these three vsiRNA is not beneficial for RNA silencing suppression and virus accumulation. Thus, we tested the binding of p20 with these three vsiRNA. It is unexpected that p20 incorporated them un-selectively. This result indicated that p20 bound sRNA in a non-sequence-specific manner.

In this paper, it was interesting to figure out the sRNA binding capacity of p20, as this is the first report of a fungal virus with an sRNA-binding RSS. However, the sRNA incorporating capability might not be the only manner for RNA silencing suppression by p20. Many genes and pathways were regulated by p20. DNA repair (methylation), RNA processing and redox regulation were the most enriched pathways among p20-regulated terms. Previously, we also found that p20 can induce H_2_ O_2_ accumulation and hypersensitive reaction in *N. benthamiana*. Future studies about the functions of p20 during host antivirus and virus counterdefense are needed.

## Data Availability

The sequencing data from this study have been submitted (http://www.ncbi.nlm.nih.gov/sra, accessed on 14 October 2022) to the NCBI Sequence Read Archive (SRA) under accession no. BioProject ID: PRJNA879941 and PRJNA879941.

## References

[1] Kondo H., Botella L., Suzuki N. (2022). Mycovirus Diversity and Evolution Revealed/Inferred from Recent Studies. Annu. Rev. Phytopathol..

[2] Kotta-Loizou I. (2021). Mycoviruses and their role in fungal pathogenesis. Curr. Opin. Microbiol..

[3] Choi G.H., Nuss D.L. (1992). Hypovirulence of chestnut blight fungus conferred by an infectious viral cDNA. Science.

[4] Hu Z., Wu S., Cheng J., Fu Y., Jiang D., Xie J. (2014). Molecular characterization of two sense-strand RNA viruses co-infecting a hypovirulent strain of *Sclerotinia sclerotiorum*. Virology.

[5] Xie J., Wei D., Jiang D., Fu Y., Li G., Ghabrial S., Peng Y. (2006). Characterization of debilitation-associated mycovirus infecting the plant-pathogenic fungus *Sclerotinia sclerotiorum*. J. Gen. Virol..

[6] Yu X., Li B., Fu Y., Jiang D., Ghabrial S.A., Li G., Peng Y., Xie J., Cheng J., Huang J. (2010). A geminivirus-related DNA mycovirus that confers hypovirulence to a plant pathogenic fungus. Proc. Natl. Acad. Sci. USA.

[7] Zhang H., Xie J., Fu Y., Cheng J., Qu Z., Zhao Z., Cheng S., Chen T., Li B., Wang Q. (2020). A 2-kb mycovirus converts a pathogenic fungus into a beneficial endophyte for brassica protection and yield enhancement. Mol. Plant..

[8] Haile J.K., N’Diaye A., Walkowiak S., Nilsen K.T., Clarke J.M., Kutcher H.R., Steiner B., Buerstmayr H., Pozniak C.J. (2019). Fusarium Head Blight in durum wheat: Recent status, breeding directions, and future research prospects. Phytopathology.

[9] Li P., Bhattacharjee P., Wang S., Zhang L., Ahmed I., Guo L. (2019). Mycoviruses in *Fusarium* species: An Update. Front. Cell Infect. Microbiol..

[10] Wang S., Kondo H., Liu L., Guo L., Qiu D. (2013). A novel virus in the family Hypoviridae from the plant pathogenic fungus *Fusarium graminearum*. Virus Res..

[11] Wang S., Zhang J., Li P., Qiu D., Guo L. (2016). Transcriptome-based discovery of *Fusarium graminearum* stress responses to FgHV1 infection. Int. J. Mol. Sci..

[12] Sioud M. (2021). RNA Interference: Story and Mechanisms. Methods Mol. Biol..

[13] Zhao J., Guo H. (2022). RNA silencing: From discovery and elucidation to application and perspectives. J. Integr. Plant. Biol..

[14] Liu Y., Teng C., Xia R., Meyers B.C. (2020). PhasiRNAs in plants: Their biogenesis, genic sources, and roles in stress responses, development, and reproduction. Plant Cell.

[15] Wang S., Li P., Zhang J., Qiu D., Guo L. (2016). Generation of a high resolution map of sRNAs from *Fusarium graminearum* and analysis of responses to viral infection. Sci. Rep..

[16] Jiang L., Wei C., Li Y. (2012). Viral suppression of RNA silencing. Sci. China Life Sci..

[17] Segers G.C., Van W.R., Zhang X., Hong Y., Nuss D.L. (2006). Hypovirus papain-like protease p29 suppresses RNA silencing in the natural fungal host and in a heterologous plant system. Eukaryot. Cell..

[18] Andika I.B., Jamal A., Kondo H., Suzuki N. (2017). SAGA complex mediates the transcriptional up-regulation of antiviral RNA silencing. Proc. Natl. Acad. Sci. USA.

[19] Sun Q., Choi G.H., Nuss D.L. (2009). A single argonaute gene is required for induction of RNA silencing antiviral defense and promotes viral RNA recombination. Proc. Natl. Acad. Sci. USA.

[20] Aulia A., Hyodo K., Hisano S., Kondo H., Hillman B.I., Suzuki N. (2021). Identification of an RNA silencing suppressor encoded by a symptomless fungal hypovirus, Cryphonectria Hypovirus 4. Biology.

[21] Yu J., Park J.Y., Heo J.I., Kim K.H. (2020). The ORF2 protein of Fusarium graminearum virus 1 suppresses the transcription of FgDICER2 and FgAGO1 to limit host antiviral defences. Mol. Plant. Pathol..

[22] Yaegashi H., Yoshikawa N., Ito T., Kanematsu S.A. (2013). Mycoreovirus suppresses RNA silencing in the white root rot fungus, *Rosellinia necatrix*. Virology.

[23] Fusaro A.F., Correa R.L., Nakasugi K., Jackson C., Kawchuk L., Vaslin M.F., Waterhouse P.M. (2012). The Enamovirus P0 protein is a silencing suppressor which inhibits local and systemic RNA silencing through AGO1 degradation. Virology.

[24] Haseloff J., Siemering K.R., Prasher D.C., Hodge S. (1997). Removal of a cryptic intron and subcellular localization of green fluorescent protein are required to mark transgenic Arabidopsis plants brightly. Proc. Natl. Acad. Sci. USA.

[25] Ruiz M.T., Voinnet O., Baulcombe D.C. (1998). Initiation and maintenance of virus induced gene silencing. Plant Cell.

[26] Melnyk C.W., Molnar A., Baulcombe D.C. (2011). Intercellular and systemic movement of RNA silencing signals. EMBO J..

[27] Huang C.Y., Wang H., Hu P., Hamby R., Jin H. (2019). Small RNAs-big players in plant-microbe interactions. Cell Host Microbe.

[28] Ghabrial S.A., Castón J.R., Jiang D., Nibert M.L., Suzuki N. (2015). 50-plus years of fungal viruses. Virology.

[29] Hillman B.I., Annisa A., Suzuki N. (2018). Viruses of plant-interacting Fungi. Adv. Virus Res..

[30] Rigling D., Prospero S. (2018). *Cryphonectria parasitica*, the causal agent of chestnut blight: Invasion history, population biology and disease control. Mol. Plant. Pathol..

[31] Min K., Neiman A.M., Konopka J.B. (2020). Shape-shifting invaders. fungal pathogens. Trends Microbiol..

